# Characterization of an Amphiphilic Exopolymer from *Rhodothermus marinus* DSM 16675 Reveals Its Potential as a Natural Bioemulsifier

**DOI:** 10.3390/ijms27198651

**Published:** 2026-09-27

**Authors:** Roya R. R. Sardari, Milad Mohammadi, Lars Dahlgren, Eva Nordberg Karlsson

**Affiliations:** Division of Biotechnology and Applied Microbiology, Department of Process and Life Science Engineering, Lund University, Naturvetarvägen 14, SE-221 00 Lund, Sweden; milad.nine@gmail.com (M.M.); lars.dahlgren@ple.lth.se (L.D.); eva.nordberg_karlsson@ple.lth.se (E.N.K.)

**Keywords:** putative glyco-lipopeptidic extracellular polymer, emulsification index, emulsifier, thermophile, *Rhodothermus marinus*

## Abstract

The thermophilic bacterium *Rhodothermus marinus* DSM 16675 represents a promising yet underexplored source of natural polymers with potential industrial applications. The aim of the current study was to screen for emulsifying activity in *R. marinus* cultures, identify conditions for production of the potential emulsifier, characterize the compound, and analyze the functional properties. Production trials in shake flasks combined with emulsification screening of the cell-free cultures showed that *R. marinus* produced an emulsifying compound, with fed-batch cultivation yielding up to 20 mg L^−1^ of crude emulsifying exopolymer. The purified exopolymer contained carbohydrates, proteins (19.22 ± 0.3 wt%), and lipids (approximately 10 wt%), with rhamnose, arabinose, and mannose as predominant carbohydrate moieties in the produced polymer. Amino acid analysis and fatty acid analyses supported its amphiphilic nature. Dynamic light scattering revealed a bimodal particle-size distribution (Z-average 288.8 ± 63.9 nm) and a moderately negative ζ-potential (−26.9 mV). The exopolymer exhibited highest emulsifying activity at 5 g L^−1^, with a critical micelle concentration (CMC) of 0.12 ± 0.01 g L^−1^ and retained good performance over pH 3–9 and 4–25 °C, whereas elevated salinity and strongly alkaline conditions reduced its activity. These findings advance knowledge on microbial exopolymers and highlight their potential as natural bioemulsifiers.

## 1. Introduction

In recent years, thermophilic microorganisms have attracted increasing attention as sources of robust biomolecules with unique structural and functional properties [1,2]. Their ability to thrive under extreme environmental conditions is reflected in their distinctive biosynthetic pathways and production of metabolites with enhanced stability and performance compared to mesophilic counterparts [3]. These characteristics make thermophiles particularly promising for industrial biotechnological applications. In the European project “SECRETed” (https://www.secreted.eu/), the aim was to screen for novel biosurfactants and/or bioemulsifiers in thermophilic microbial species and connect this activity to specific molecules in targeted microorganisms. *R. marinus* DSM 16675 was identified as a producer of the desired activity and selected for further investigation, purification and characterization of the compound(s) responsible.

*Rhodothermus marinus* DSM 16675 is a moderately halophilic and thermophilic bacterium originally isolated from marine hot springs [4]. It has emerged as a valuable candidate for biorefinery processes due to its capacity to utilize second- and third-generation feedstocks, supported by a diverse repertoire of biomass-degrading enzymes [5]. In addition to enzymes, *R. marinus* produces a range of biotechnologically interesting metabolites, including sulfated exopolysaccharides (EPSs) and carotenoids [6,7]. However, functional characterization has not yet been in focus, and in this work surface-active functionality was targeted, combined with identification and characterization of the extracellular molecules resulting in this activity.

Extracellular polymers have gained considerable attention due to the growing demand for natural polymers across various industries. They are high-molecular-weight biopolymers secreted into the extracellular environment, where they contribute to microbial survival by mediating adhesion, protection, and interactions with surrounding interfaces [3]. While extracellular polymers are sometimes described as exopolysaccharides, microbial extracellular polymers are often structurally more complex, comprising not only carbohydrates but also significant fractions of proteins and lipids. Such heterogeneous extracellular polymers may exhibit amphiphilic characteristics, allowing them to stabilize oil-water interfaces and function as emulsifiers. Bioemulsifiers (emulsifiers originating from living organisms) are particularly relevant for stabilizing emulsions formed between immiscible phases such as water and hydrophobic compounds. These emulsion systems are inherently unstable and prone to processes such as coalescence, flocculation, and phase separation, necessitating use of stabilizing agents [8].

In contrast to low-molecular-weight surfactants, microbial bioemulsifiers are typically high-molecular-weight heteropolymers—including glycoproteins, lipopolysaccharides, and lipopeptide-containing polysaccharides—that combine hydrophilic and hydrophobic moieties, enabling effective interfacial stabilization [9]. Compared to synthetic emulsifiers, bioemulsifiers offer several advantages such as biocompatibility, biodegradability, lower toxicity, effectiveness at low concentrations and functionality under a wide range of environmental conditions. Stability across varying temperatures, pH levels, and ionic strengths further enhances their suitability for diverse food applications [10], but also for pharmaceuticals, cosmetics, detergents, environmental remediation and other industrial sectors [8].

This work resulted in the identification of an extracellular polymer with emulsifying activity, and was followed by compositional analysis of its carbohydrate, protein, and lipid components together with physicochemical characterization of its emulsifying properties under different environmental conditions. This study provides new insight into the composition and functionality of the emulsifying extracellular polymer produced by *R. marinus* DSM 16675 and highlights its potential as a natural bioemulsifier for biotechnological and industrial applications.

## 2. Results

### 2.1. Production of Exopolymer with Emulsifying Activity

Growth and production of emulsifying compounds by *R. marinus* DSM 16675 in shake flasks were evaluated in media 162 and MB under batch and single-cycle fed-batch conditions. While both media and both modes of operation supported growth, only fed-batch cultivation resulted in significant emulsifying activity. The culture broths from 162 and MB media exhibited similar emulsification index (E_24_) of 60.7% and 64.3%, respectively, despite the considerably lower exopolymer production in 162 medium (Table 1). Although both media supported similar emulsifying activity, MB medium resulted in a substantially higher exopolymer yield compared to 162 medium. Therefore, MB medium was selected due to providing a higher exopolymer yield without compromising emulsifying activity, facilitating the recovery of sufficient material for subsequent experiments.

In a previous study, Ron et al. (2019) [11] reported exopolysaccharides yields of 3.77 and 19 mg/L during sequential batch cultivations of *R. marinus* DSM 16675 in LB and MB media supplemented with maltose (LB_malt_ and MB_malt_), respectively. Similarly, Mukti et al. (2022) [12] reported an exopolysaccharide yield of 3.4 ± 0.17 mg/L during batch cultivation of the same strain in a defined medium. These previously reported yields are comparable to those obtained in the present study (3.83 and 19.89 mg/L; Table 1). However, the results were obtained from independent experiments conducted under different culture media and cultivation conditions.

### 2.2. Composition Analysis Revealing a Putative Glycol-Lipopeptidic Polysaccharide Structure

Following hydrolysis of 3.6 mg of the purified exopolymer, five monosaccharides were quantified: glucose, galactose, mannose, arabinose, and rhamnose. The total measured amount of these monosaccharides was 0.162 mg, corresponding to 5.40 wt% of the hydrolyzed sample. The purified exopolymer exhibited hetero-polysaccharidic carbohydrate content dominated by rhamnose, arabinose and mannose (Table 2). Such monosaccharide profiles are consistent with the structurally diverse nature of microbial exopolysaccharide, which commonly occur as heteropolysaccharides with variable sugar compositions. This compositional heterogeneity is known to influence physicochemical behavior and may contribute to intermolecular interactions relevant to emulsion stabilization [13]. It is also a composition that differs in carbohydrate moieties and ratios from the previously isolated EPS from *R. marinus*, where the EPS contained significant amounts of xylose [6], but where cultivation conditions differed.

A substantial protein fraction (19.22 ± 0.29% *w*/*w*) was also detected (Table 3), confirming that the polymer is not purely polysaccharidic, but including a glycoprotein-rich matrix. Glycine (2.13 ± 0.15) and L-serine (2.02 ± 0.24) were the most abundant amino acids, while L-cysteine (0.07 ± 0.03) was only present in low amounts, indicating a composition potentially favorable for solubility and flexibility. Moreover, the presence of hydrophobic amino acids (e.g., L-Leucine, L-Phenylalanine, L-Valine) contributes to the amphiphilic behavior, which is critical for emulsification.

Fatty acid analysis was performed on the purified exopolymer following two-phase extraction with 0.15 M aqueous acetic acid and methyl tert-butyl ether (MTBE), followed by the methylation of lipids that were covalently bound or physically associated with the exopolymer. The total lipid content was estimated to be approximately 10 wt% of the purified exopolymer, with the estimated lipid mass distributed approximately equally between the aqueous and organic fractions, although the profiles differed between the two fractions. This can be seen in the extracted ion chromatogram, which was prepared for *m*/*z* = 74.1 (Figure 1B), as this is a characteristic mass fragment for the McLafferty rearrangement ion of methyl esters [14].

The dominant fatty acids had retention times between 5 and 10 min, and to identify the lipids, positive identification was made by matching retention time with the external standard (Figure 1A) and by comparing with library mass spectra. The identified fatty acids included lauric acid (C12:0), myristic acid (C14:0), pentadecanoic (C15:0), palmitic acid (C16:0), palmitoleic acid (C16:1), stearic acid (C18:0), and oleic acid (C18:1). The aqueous fraction contained multiple unidentified fatty acids (A–E, G, Figure 1B) with retention times between those of lauric acid and oleic acid. These aqueous fraction lipids are judged to be esterified to hydrophilic (polar) moieties preventing extraction into MTBE, despite their long-chain fatty acid moiety. The organic fraction lipids represent lipids enclosed in or non-covalently bound to the exopolymer that could be released and extracted by MTBE.

Peaks A–E and G in the aqueous fraction could not be assigned to specific compounds. Their contribution to the estimated lipid content was calculated using the average GC-FID response factor of the GLC-426 fatty acid methyl ester (FAME) standard. Because the FID response depends on the amount of carbon reaching the detector, this approach provides an approximate mass estimate but does not permit the accurate quantification of unidentified compounds. More precise quantification would require the identification of peaks A–E and G and the determination of compound-specific response factors, preferably using authentic standards.

Together, the coexistence of carbohydrate, protein, and lipid fractions suggests a putative glyco-lipopeptidic exopolymer structure with intrinsic amphiphilic architecture, explaining its surface-active properties.

### 2.3. Determination of Emulsifying Properties

#### 2.3.1. Emulsification Activity and Emulsion Stability

The emulsification activity and emulsion stability increased markedly with the increase in crude exopolymer concentration (Table 4), supporting that this polymer is responsible for the emulsifying properties. At 5 g/L, the system reached its highest performance, with strong initial activity and with sustained emulsion stability over 120 h (88%).

Purified exopolymer at 5 g/L exhibited a slightly lower initial emulsification activity (40% at 1 h) than the crude variant, but demonstrated remarkably long-term stability, retaining nearly 100% of its initial activity after 24 h, 120 h, and even after several weeks. This suggests that non-polymeric components in the crude extract may enhance initial emulsification, while the purified polymer provides structural stabilization.

Due to the limited availability of the exopolymer, the concentration-dependent emulsification assays using the crude exopolymer at different concentrations and the purified exopolymer at 5 g/L, as presented in Table 4, were performed only once as preliminary results. Based on these preliminary results, a concentration of 5 g/L crude exopolymer was selected for further characterization of its emulsification properties under different pH, temperature, and salinity conditions. These experiments were performed in triplicate.

#### 2.3.2. Effect of pH

The emulsification activity of the exopolymer showed some pH dependence (Figure 2), decreasing to zero at highly alkaline conditions. The activity was tested at pH (3, 5, 7, 9, 11) after 1, and 24 h. The highest emulsification activity was observed under neutral to mildly alkaline conditions (pH 7–9), with the highest emulsion stability recorded at pH 9 (47.27 ± 2.57%, after 1 h, and unchanged after 24 h). The emulsification activity at pH 7 was the same after 1 h but decreased to 43.64 ± 2.57% after 24 h.

In acidic conditions (pH 3–5), a small reduction in emulsification index was noticed, but the most pronounced reduction was observed at pH 11, where the emulsification index decreased to 27.27± 0.0% after 1 h and was completely lost after 24 h. Additionally, acidic conditions at pH 3 and 5 had a small adverse effect on the emulsification activity, leading to a loss of 11.11% and 10.64% of the activity, respectively, after 24 h. These results indicate that ionizable functional groups within the polymer play a role in interfacial stabilization.

#### 2.3.3. Effect of Temperature

Temperature influenced both emulsification activity and emulsion stability (Figure 3). The exopolymer showed optimal emulsification activity at 25 °C (56.67 ± 4.71%) and retained comparable activity at 4 °C (55 ± 7.07%), demonstrating functionality across a broad temperature range. A further increase in temperature (in the range 50 to 100 °C) led to a progressive decline in the activity, with complete loss at 100 °C (Figure 3). These findings suggest that the polymer exhibits moderate thermal tolerance, as its interfacial structure is affected at elevated temperatures.

#### 2.3.4. Effect of Salinity

The effect of salinity (NaCl) on the emulsification activity and emulsion stability was investigated at different NaCl concentrations (1, 3, 5, and 10%) and was shown to have a pronounced negative effect on the emulsification of the crude exopolymer (Figure 4). While moderate activity was retained at 1% NaCl (52.73 ± 0.0% after 1 h, it decreased to 45.45 ± 0.0% after 24 h). In addition, an increasing salt concentration resulted in a sharp decline in emulsification activity (27.27 ± 12.86% at 3% NaCl, and further to 4.55 ± 1.29% at 5% NaCl, with no remaining activity after 24 h), and with complete loss of emulsification activity ≥5% NaCl. This sensitivity suggests that electrostatic interactions are important, as high ionic strength leads to destabilization of the emulsion.

### 2.4. Colloidal Properties and Particle Behavior

The hydrodynamic properties of the exopolymer were also analyzed. Dynamic light scattering (DLS) revealed a Z-average diameter of 288.8 ± 63.9 nm with a polydispersity index (PDI) of 0.381 ± 0.025, indicating a moderately heterogeneous particle distribution (Table 5). The intensity-based size distribution showed a bimodal pattern [15], with particle populations detected in the approximate ranges of 80–120 nm and 290–374 nm (Supplementary Figure S1). The ƺ-potential was (−26.88 mV) which indicates a moderate negative surface charge, suggesting the presence of ionizable groups and providing moderate colloidal stability.

It should be noted that an average molecular weight of approximately 80 kDa was reported in our previous study for the exopolymer produced by *R. marinus* DSM 16675, which was previously described as an exopolysaccharide [6].

### 2.5. Critical Micelle Concentration (And Self-Assembly)

The fluorescent emission spectra of the aqueous purified exopolymer solutions at concentrations between 0.02 and 0.5 g/L was recorded at room temperature, using curcumin as a fluorescence probe at λex = 425 nm (Figure 5). Emission intensities were plotted against the concentration for each sample. Although concentrations ranging from 0.01 to 5 g/L were evaluated, Figure 5 presents only the 0.02–0.5 g/L range to allow clearer visualization of the change used to determine the CMC.

Curcumin has an emission peak at 553 nm [16] which was shifted to 500 nm in different concentrations of exopolymer and thus, the emission wavelength 500 nm was used for critical micelle concentration (CMC) determination. This shift indicates incorporation of the probe into a hydrophobic microenvironment, consistent with micelle formation. This shift was suggested by Zakaria and coworkers [17] to be due to the nonpolar micellar environment created by the investigated sample. Emission intensities were plotted against purified exopolymer concentrations (Figure 5). When a curcumin probe is exposed to an aqueous solution, it exhibits a weak fluorescence signal. As the polymer concentration increased, the curcumin probe was solubilized in the hydrophobic core of the formed micelles and the fluorescence increased [18]. The CMC was calculated from the intersection of the two linear regressions (y = ax + y0) at high and low concentrations of the exopolymer. The CMC was determined to be 0.12 ± 0.01 g/L, indicating that the polymer can self-assemble into micellar structures at relatively low concentrations. This behavior provides further evidence for its amphiphilic nature and explains its efficiency as a bioemulsifier.

## 3. Discussion

This study provides a comprehensive physicochemical characterization of the extracellular polymer produced by *Rhodothermus marinus* DSM 16675, with particular emphasis on its composition–function relationships and emulsifying properties. The results demonstrate that cultivation conditions strongly influence exopolymer production. The combination of batch cultivation followed by a stepwise single-cycle fed-batch process in MB medium improved production (20 mg/L), indicating that both time and nutrient availability (requiring a feeding strategy) are critical determinants for polymer synthesis. Although the overall yield remained moderate, the produced exopolymer exhibited a high emulsification index, highlighting that functional performance rather than quantity alone is a key advantage of thermophilic microbial polymers [19], and is highlighting their potential for applications in biotechnological and industrial processes.

The composition analysis of the purified exopolymer identified rhamnose as the predominant sugar, followed by arabinose. Rhamnose (6-deoxy-L-mannose) features a methyl group (-CH_3_) at the C-6 position instead of a hydroxyl group (-OH), which decreases its polarity and increases hydrophobicity compared to other sugars. Exopolymers rich in rhamnose may exhibit amphiphilic characteristics, containing both hydrophilic and hydrophobic regions that enable alignment at oil–water interfaces, functioning similarly to surfactants in stabilizing emulsions. The presence of arabinose is particularly notable, as it is rarely found in microbial exopolymers and may indicate unique biosynthetic pathways in the producing strain. Due to its multiple hydroxyl groups, arabinose is highly hydrophilic and contributes to enhanced water solubility and the formation of a hydrated interfacial layer around oil droplets, helping to stabilize emulsions by preventing droplet coalescence [20].

The amino acid composition of the exopolymer revealed a significant proteinaceous component (19.2%). The combination of protein and a high negative charge may act synergistically to enhance the emulsifying capacity of the exopolymer for mineral oil [21]. The amino acid profile rich in glycine, serine, arginine, and lysine suggests the presence of glycoproteins with potential structural flexibility, metal-binding capacity, and glycosylation sites. Glycine may contribute to conformational flexibility because of its small side chain [22] while serine contains a hydroxyl group that can serve as a glycosylation site [23]. The charged side chains of arginine and lysine may also participate in interactions with other components of the exopolymer [24]. These potential roles could influence the organization and interfacial properties of the exopolymer; however, the specific contributions of these amino acids remain to be established.

The distribution of lipids between aqueous and organic fractions in exopolymers has functional implications, particularly in their role as bioemulsifiers. Lipids in the aqueous fraction may be associated with hydrophilic groups, enhancing solubility and interaction with polar environments, while those in the organic fraction may contribute to hydrophobic interactions [25].

Compositional analysis of the purified exopolymer revealed 5.4 wt% identified monosaccharides, 19.2 wt% protein, and approximately 10 wt% lipid, collectively accounting for 35.4% of the sample mass. The remaining 64.6% could not be assigned to specific constituents based on the analyses performed. Similarly, low measured carbohydrate content and incomplete compositional mass balances have been reported for other marine bacterial exopolymers. For example, the surface-active exopolymer produced by *Halomonas* sp. TGOS-10 was reported to contain 1.5 wt% carbohydrate and 46.6 wt% protein, with more than 50% of its mass remaining unaccounted for [26]. The authors suggested that resistance to acid hydrolysis might have contributed to the low measured carbohydrate content.

In the present study, uronic acids were not detected. Sulfate groups potentially associated with the exopolymer, as well as residual salts that may have remained after purification, could contribute to the unassigned fraction (64.6%). The presence of sulfate groups may also contribute to the negative ζ-potential (−26.9 mV) observed for the exopolymer. Further analyses, including ash and sulfate content determinations, are required to clarify the composition of the unassigned fraction.

The emulsification activity and stability of the crude exopolymer were evaluated. The results showed that the effectiveness of the exopolymer as bioemulsifier depends on concentration. Emulsification activity increased to 50% at 4 g/L and 57% at 5 g/L, with greater emulsion stability observed at 5 g/L. Since emulsions are thermodynamically unstable systems, maintaining their stability remains a significant challenge [27,28], and different conditions were applied to evaluate the performance. Environmental conditions (pH, temperature and salinity) had a pronounced effect on emulsion behavior, further supporting the importance of electrostatic and structural factors.

Optimal performance was observed at neutral to slightly alkaline pH, where the polymer is predominantly negatively charged. This promotes electrostatic repulsion between droplets, preventing aggregation and enhancing stability. In contrast, acidic conditions reduce charge density through protonation, while extreme alkalinity may disrupt glycosidic linkages, both leading to decreased performance [29]. At pH 9, the exopolymer was predominantly deprotonated and negatively charged. This negative charge enhanced electrostatic repulsion between exopolymer molecules adsorbed at the oil–water interface, preventing droplet coalescence and stabilizing the emulsion. At pH 7, the charge was also present but less pronounced (closer to neutrality), which might explain the slight decrease in stability (43.64 ± 2.57% after 24 h) due to weaker repulsion. As stated above, extreme alkalinity (pH 11) might break glycosidic bonds in the exopolymer backbone, reducing molecular weight and emulsifying capacity. In acidic conditions (pH 3 or 5), the exopolymers were protonated, reducing repulsion and solubility, leading to the observed activity losses (11.11% and 10.64%, respectively) [30].

Temperature effects revealed that the exopolymer maintains functionality at low to moderate temperatures but loses activity at elevated temperatures. This behavior is likely associated with temperature-induced changes in viscosity and the weakening of interfacial interactions. Over time, temperature variations affected emulsion stability in different ways. The loss of activity at higher temperatures was likely attributed to a decrease in viscosity, resulting in emulsion breakdown. Elevated temperatures disrupted interactions between exopolymer molecules and the emulsion interface, which is earlier reported to lead to phase separation [31]. While this may be a limitation for applications requiring long-term emulsion stability, it can be advantageous in processes where controlled emulsion destabilization is needed.

Salinity was identified as a critical limiting factor. Increasing NaCl concentrations led to a rapid decline in emulsifying activity, which can be attributed to charge screening and polysaccharide aggregation. A marked decrease in activity was observed when the salt concentration exceeded 1%. Elevated salt levels facilitated the aggregation of exopolymer molecules, which reduces the surface area available for stabilizing the emulsion, resulting in decreased stability. Furthermore, higher salinity accelerates the coalescence of oil droplets, causing the formation of larger droplets and making the emulsion less stable [32]. It is important to note that the salinity-dependent emulsification experiments were conducted using the crude exopolymer, while the purified fraction was evaluated separately for its emulsifying properties and long-term emulsion stability. In the absence of added salt, the purified exopolymer (5 g/L) maintained an emulsification index of approximately 40% over several weeks, demonstrating its ability to form and stabilize emulsions independently of additional components present in the crude preparation. However, the salt tolerance of the purified exopolymer cannot be directly inferred from the results obtained with the crude fraction, as other components present in the crude preparation may influence its emulsifying behavior. Therefore, the present findings provide a preliminary assessment of the exopolymer’s functional properties and potential industrial applicability. Further investigation of the purified fraction under different ionic-strength conditions would be necessary to determine its suitability for specific industrial formulations.

Colloidal characterization further supported the functional observations. The molecular size distribution and aggregation behavior of the crude exopolymer were analyzed using DLS. The combination of high hydration, potential aggregation, and a strong negative charge suggest that the exopolymer may have favorable rheological and emulsifying properties [33]. The charge stability of exopolymer can enhance their dispersibility in aqueous solutions by maintaining a consistent surface charge, preventing unwanted aggregation or precipitation, making them highly suitable for applications in food, pharmaceuticals, and environmental biotechnology [34,35].

The ability of the exopolymer to form micelle-like structures, as demonstrated by fluorescence spectroscopy using curcumin, provides additional insight into its mode of action. The results showed that the fluorescence intensity of curcumin increased and exhibited a spectral shift in the exopolymer system, demonstrating the potential of curcumin as a fluorescent probe for studying exopolymer aggregation behavior. This approach highlighted the amphiphilic characteristics of the exopolymer and their potential for applications involving self-assembly and emulsion stabilization.

The relatively low critical micelle concentration (CMC = 0.12 ± 0.01 g/L) indicates efficient self-assembly and significant surface activity, despite the polymeric nature of the system. Although structurally distinct from conventional low-molecular-weight surfactants, the combination of carbohydrate, protein, and lipid components enables this exopolymer to function as an effective bioemulsifier through multivalent interactions and cooperative interfacial organization via presence of amphiphilic functional groups [34].

Overall, the findings demonstrate that the extracellular polymer produced by *R. marinus* DSM 16675 suggests a putative glyco-lipopeptidic exopolymer with promising bioemulsifying properties. Its ability to stabilize emulsions, combined with its marine thermophilic origin, highlights its potential as a natural and sustainable alternative to synthetic emulsifiers in biotechnological and pharmaceutical applications.

## 4. Materials and Methods

The Difco™ Marine Broth 2216 was purchased from BD (Franklin Lakes, NJ, USA), and ethanol absolute (99.5% pure, analytical grade) from Solveco (Rosersberg, Sweden). All the other chemicals were either analytical grade or LC-MS grade and purchased from Merck (Sigma-Aldrich, Stockholm, Sweden) unless otherwise mentioned.

### 4.1. Bacterial Strain and Medium Preparation

In the present study, *Rhodothermus marinus* DSM 16675 was used. Two different complex media Marine Broth 2216 (MB), and modified Thermus 162 medium with 2% NaCl (162) according to DSMZ specifications [36] were employed, separately.

### 4.2. Inoculum Preparation

The inoculum was prepared by mixing 1 mL stock culture (kept at −80 °C in cryotube with 25% *v*/*v* glycerol) to 25 mL of the desired medium in a 250 mL baffled shake flask. The culture medium was then incubated at 65 °C and 200 rpm for 18 h (OD ≥ 2, first step of inoculum preparation). In the second step, 5 mL of the culture from step one was transferred to a 500 mL baffled shake flask containing 45 mL of the desired medium (10% *v*/*v* inoculum) and incubated at 65 °C and 200 rpm for 12 h (OD ≥ 2, second step of inoculum preparation). The resulting culture was used as inoculum (10% *v*/*v*) for further cultivation, while a part of the inoculum was left during 24 h for subsequent screening of emulsifying activity.

### 4.3. Screening Strategies for Emulsifying Activity and Exopolymer Production

A portion of the inoculum culture supernatant was added to an equal-volume test oil (as described under Section 4.6.1 and Section 4.6.2) to verify the presence of emulsifying activity. After positive screening results, a total of four *R. marinus* DSM 16675 cultivation strategies were then investigated to evaluate production of emulsifying compounds. These strategies were divided by their mode of operations, into two batch cultivations and two single-cycle stepwise fed-batch cultivations. All these cultivations were performed in 1 L baffled shake flasks containing 100 mL of the desired medium with 10% *v/v* inoculum. The cultures were incubated at 65 °C and 200 rpm. For batch cultivations, the cultures were incubated for 24 h. For fed-batch cultivations, the cultures were initially grown in batch mode for 12 h, after which 50 mL of fresh medium (as described in Section 4.1) was added stepwise to each shake flask as a single feeding event. Cultivation was then continued for an additional 24 h, resulting in a total cultivation time of 36 h.

### 4.4. Isolation and Purification of Exopolymers from the Cell-Free Culture Broth

The cell-free culture broth was obtained from the centrifugation of the culture broth twice for 10 min at 3893× *g* (Sorvall LYNX 4000, ThermoFisher Scientific, Waltham, MA, USA). After that, ethanol (99%) was added to the culture broth with a ratio of 1:4 (broth: ethanol *v*/*v*), and the mixture was incubated at 4 °C overnight. The resulting precipitate, a crude glycan-rich exopolymer fraction, was then collected by centrifugation of the mixture for 10 min at 3893× *g* and open-air dried. The crude exopolymers obtained were fractionated using size-exclusion chromatography as described previously by Sardari et al. [6] to obtain a pure fraction.

### 4.5. Composition Analysis

#### 4.5.1. Carbohydrate Composition Analysis

The carbohydrate composition of the purified exopolymers was analyzed following a modified version of the method described by Sluiter et al. [37]. Briefly, 175 µL of sulfuric acid (72% *w*/*w*) was added to 3 mg of purified exopolymer in a glass tube and incubated in a heating block at 30 °C for 1 h. Afterward, 4.9 mL of ultrapure water (MilliQ grade) was added to dilute the sulfuric acid concentration to 4% (*w*/*w*). The samples were then incubated at 100 °C for 3 h using a Techne Dri-Block^®^ Digital Block Heater (Bibby Scientific Ltd., Stone, Staffordshire, UK), followed by cooling and neutralization with 0.1 M barium hydroxide. Following centrifugation, the monosaccharide content in the supernatant was analyzed using high-performance anion exchange chromatography with pulsed amperometric detection (HPAEC-PAD, Thermo Fisher Scientific, Waltham, MA, USA). The analysis was performed with a Dionex CarboPac PA-20 guard column coupled to a Dionex CarboPac PA-20 analytical column. Ultrapure water (pump A), 2 mM NaOH (pump B), and 200 mM NaOH (pump C) were used as eluents, delivered through three separate pumps. Separation was achieved within a 23 min runtime using an isocratic flow of 0.5 mL/min with a mixture of 62.5% of ultrapure water and 37.5% of 2 mM NaOH. The column and compartment temperatures were maintained at 30 °C. The column was regenerated after each run with 200 mM NaOH for 2 min. Monosaccharides were detected using an ED40 electrochemical detector (Dionex Corporation, Sunnyvale, CA, USA).

Uronic acids were analyzed under isocratic conditions at a flow rate of 0.5 mL/min for 30 min, with the column and compartment temperatures maintained at 30 °C. The mobile phase consisted of 55% ultrapure water (pump A), 15% of 1 M sodium acetate (pump B), and 30% of 200 mM NaOH (pump C). Galacturonic acid, Glucuronic acid, guluronic acid, and mannuronic acid were used as standards.

#### 4.5.2. Amino Acid Composition Analysis

The amino acid composition of the purified exopolymers was analyzed after hydrolysis using a method described by Sardari et al. [38]. Liquid-phase acid hydrolysis was conducted in Pyrex glass tubes, where 1 mg of the sample was mixed with 1 mL of 6 M HCl (amino acid analysis grade, Merck, Sweden) containing 0.1% (*w*/*v*) phenol. The tubes were flushed with nitrogen before sealing and incubated at 110 °C for 24 h in Techne Dri-Block^®^ Digital Block Heater (Bibby Scientific Ltd., UK). Following hydrolysis, the acid was removed using the Techne sample concentrator (Cole-Parmer, Stone, Staffordshire, UK), and the amino acids were reconstituted in 1 mL of ultrapure water before analysis. Quantitative determination of amino acid composition was performed using high-performance anion-exchange chromatography (HPAEC-PAD, ThermoFisher Scientific, Waltham, MA, USA). The analysis was carried out with a Dionex AminoPac PA10 analytical column coupled to a Dionex AminoPac PA10 guard column. The HPAEC system operated with four eluents: ultrapure water (pump A), 1 M sodium acetate (pump B), 0.25 M NaOH (pump C), and 0.1 M acetic acid (pump D). Separation was achieved over a 74 min runtime under gradient conditions at a flow rate of 0.25 mL/min. Detection was performed using an ED40 electrochemical detector. Before injection into the Dionex system, samples were filtered through a 0.2 μm polypropylene filter. A TraceCERT-grade certified reference amino acid mix (Merck (Sigma-Aldrich), Stockholm Sweden) was used for standardization.

#### 4.5.3. Fatty Acid Composition Analysis

The fatty acid composition of purified exopolymers was determined by gas chromatography mass spectrometry (GC-MS). Two milligrams purified exopolymer fraction was dissolved in 2 mL 0.15 M acetic acid (aq). This solution was extracted twice by addition of 2 mL tert-butyl methyl ether (MTBE) followed by vortexing and centrifugation. The top (organic) fractions were collected and pooled. Both the organic and aqueous fractions were then dried under nitrogen flow at ambient temperature until dryness. The samples were resuspended in 2% H_2_SO_4_ in methanol and methylated at 80 °C for 45 min. Then the samples were cooled, and 2 mL ultrapure water and 2 mL heptane were added followed by vortexing and centrifugation, after which the top (organic) fractions were collected. These fractions were then dried under nitrogen flow at ambient temperature, resuspended in 150 µL heptane, and transferred to glass vials for analysis.

Analysis was performed using a ISQ7610 single quadropole MS coupled to a Trace 1300 GC equipped with an AS 1310 autosampler (Thermo Scientific, Waltham, MA, USA). The extracted fatty acid methyl esters were separated on a Zebron ZB-Fame capillary GC column with the length 20 m, inner diameter 0.18 mm, and film thickness 0.15 µm (Phenomenex, Torrance, CA, USA). Analysis was performed using a ramped temperature program starting at 80 °C, held for 4 min, followed by a 40 °C/min gradient until 160 °C was reached. The gradient was then decreased to 5 °C/min until 260 °C was reached.

Injection was performed in split mode at a 10:1 split ratio. The injector, detector transfer line, and ion source temperatures were set to 250 °C. The ionization energy was set to 70 eV, and the MS ran for full-scan acquisition ranging from 30 to 400 m/z. Identification of fatty acid methyl esters was performed by comparison to the external standard GLC-426 (Nu-Chek Prep, Elysian, MN, USA) and by comparison to NIST (version 17) library spectra.

The lipid content of the purified exopolymer was estimated by GC-FID using response factors obtained from external standards for the identified fatty acids. For fatty acids that could not be matched to the external standards, their contribution to the estimated lipid content was calculated using the average GC-FID response factor of the GLC-426 fatty acid methyl ester (FAME) standard. As the FID response is proportional to the amount of carbon reaching the detector, this approach provides an approximate estimate of their mass.

### 4.6. Evaluation of Emulsification Activity and Emulsion Stability of Purified Fractions

#### 4.6.1. Emulsion Preparation

Emulsions of exopolymer fractions were prepared with equal volumes of aqueous solutions of the polymer fraction and mineral oil. The two phases were manually shaken and vortexed for 5 min, then allowed to stand at room temperature for the specified duration.

#### 4.6.2. Determination of Emulsification Activity

The emulsification activity of the isolated exopolymers was assessed by preparing different concentrations of the crude exopolymers (1–5 g/L), and 5 g/L of purified exopolymer. The emulsification activity of the samples (Equation (1)) was determined after 1, 24, and 120 h while the emulsion stability (Equation (2)) was evaluated after 24 h and 120 h at room temperature. A control containing the emulsification assay mixture without added exopolymer was prepared under the same conditions.

(1) $$Emulsification\;activity=\left(Height\;of\;emulsion\;layer/Total\;hight\;of\;the\;mixture\;\right)\times 100$$

*Emulsion stability* = (*Final activity*/*Initial activity*) × 100 (2)

Final activity refers to the emulsification activity measured after a certain period. Initial activity refers to the emulsification activity measured after 1 h.

#### 4.6.3. Effect of pH on Emulsion Stability

The effect of pH was investigated by dissolving crude exopolymers (5 g/L) in sodium phosphate buffer solution (0.05 M) with different pH values (3, 5, 7, 9, 11). The pH values were adjusted to the desired values using hydrochloric acid (1 M) and sodium hydroxide (1 M). The emulsions were then prepared and stored at room temperature for 1 and 24 h, after which their emulsification activity was measured.

#### 4.6.4. Effect of Temperature on Emulsion Stability

The effect of temperature was investigated by dissolving crude exopolymers (5 g/L) in ultrapure water. The emulsions were prepared in Pyrex glass tubes sealed with closed-top screw caps fitted with preassembled septa and maintained at 4, 25, 50, 80, or 100 °C for 1 or 24 h, after which their emulsification activity was measured.

#### 4.6.5. Effect of Salinity on Emulsion Stability

The effect of salinity was investigated by dissolving crude exopolymers (5 g/L) and NaCl with the concentration of (1, 3, 5, 10%) in ultrapure water. The emulsions were then prepared and stored at room temperature for 1 and 24 h before measuring their emulsification activity.

### 4.7. Determination of Hydrodynamic Diameter and ƺ-Potential by Dynamic Light Scattering (DLS)

An aqueous solution of crude exopolymers (0.1 g/L) was prepared in ultrapure water. The hydrodynamic diameter and ƺ-potential of the exopolymer were measured using a dynamic light scattering (DLS) apparatus (Zetasizer Nano ZS, Malvern Instruments, Malvern, UK). The crude exopolymer was dispersed in ultrapure water and analyzed at 25 °C. Hydrodynamic diameter measurements were performed in triplicate (*n* = 3), whereas ζ-potential was measured once (*n* = 1).

### 4.8. Determination of Critical Micelle Concentration (CMC)

The CMC of the purified exopolymer fraction was determined by fluorescence using 1,7-bis(4-Hydroxy-3-methoxyphenyl)-1,6-heptadiene-3,5-dione (curcumin) as a fluorescence probe (14, 16). A stock solution of the fluorescence probe (2 g/L) was prepared by adding 10 mg of curcumin (analytical standard, Merck (Sigma-Aldrich), Stockholm, Sweden) in 5 mL ethanol solution (99%) followed by bath sonication until a clear solution was achieved. A working solution of curcumin (0.1 mM) was prepared by diluting 184 μL of the stock curcumin solution in ultrapure water to a final volume of 10 mL which was used for further dilutions. Different samples with exopolymer concentrations ranging from 0.01 to 5 g/L containing a fixed curcumin concentration of 2 μM were subjected to fluorescence measurements using 200 μL per well of a 96-well black plate. The emission spectra were recorded at 25 °C using a spectrofluorometer (BioTek Synergy H1 hybrid microplate reader, Agilent Technologies, Inc., Santa Clara, CA, USA) at wavelengths ranging from 460 to 700 nm. The wavelength of excitation was fixed at 425 nm and the measurements were taken in duplicate.

## 5. Conclusions

This study demonstrates that *Rhodothermus marinus* DSM 16675 produces a structurally complex extracellular polymer with pronounced emulsifying properties when cultivated under fed-batch conditions. Compositional and functional analyses revealed that this material is not a simple polysaccharide but an exopolymer with putative glyco-lipopeptidic structure, whose amphiphilic architecture—arising from carbohydrate, protein, and lipid components—underpins its interfacial activity. The crude exopolymer exhibited promising emulsifying properties, with activity and stability influenced by concentration and physicochemical conditions, including pH, temperature, and salinity. Notably, colloidal characterization of the crude exopolymer revealed a hydrodynamic diameter of 288.8 ± 63.9 nm and a negative ζ-potential of −26.88 mV, providing further insight into its physicochemical properties. However, challenges remain, including reduced emulsifying activity of the crude exopolymer at elevated temperatures and high salinity (>1% NaCl), which may limit its use in certain applications. The purified exopolymer (5 g/L) maintained an emulsification index of approximately 40% over several weeks in the absence of added salt, confirming its ability to stabilize emulsions independently of additional components present in the crude preparation. Fluorescence spectroscopy using curcumin revealed the amphiphilic nature of the purified exopolymer, with a low critical micelle concentration (0.12 ± 0.01 g/L), highlighting its potential for self-assembly and colloidal applications. Further investigations of the purified exopolymer under varying environmental conditions are necessary to establish its functional performance and suitability for specific industrial applications.

Overall, the results demonstrate the potential of this marine-derived exopolymer as a natural bioemulsifier.

## Figures and Tables

**Figure 1:**
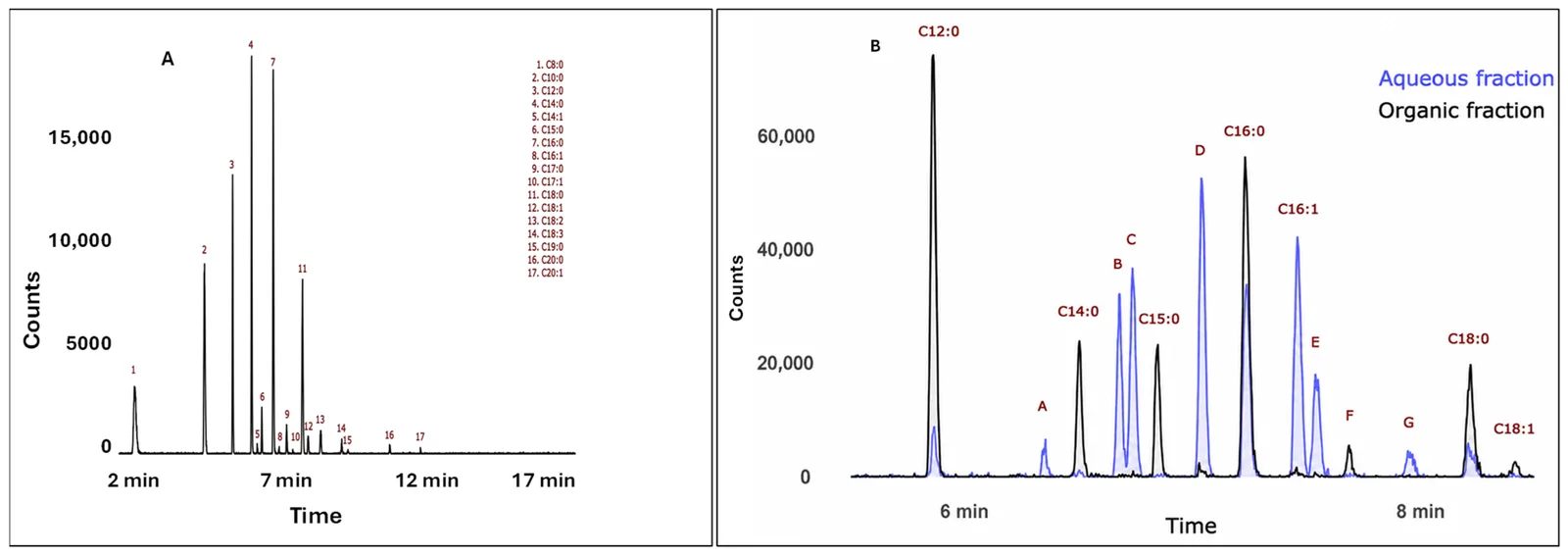
(**A**) Representative GC–MS chromatogram of fatty acid methyl esters (FAMEs). Peaks correspond to the identified fatty acids. (**B**) Extracted ion chromatogram *m*/*z* = 74 for organic (black) and aqueous (blue) fraction. Identified fatty acid methyl esters marked with their respective carbon chain length and degree of unsaturation. Fatty acid methyl esters not corresponding to the external standard are marked A–G.

**Figure 2:**
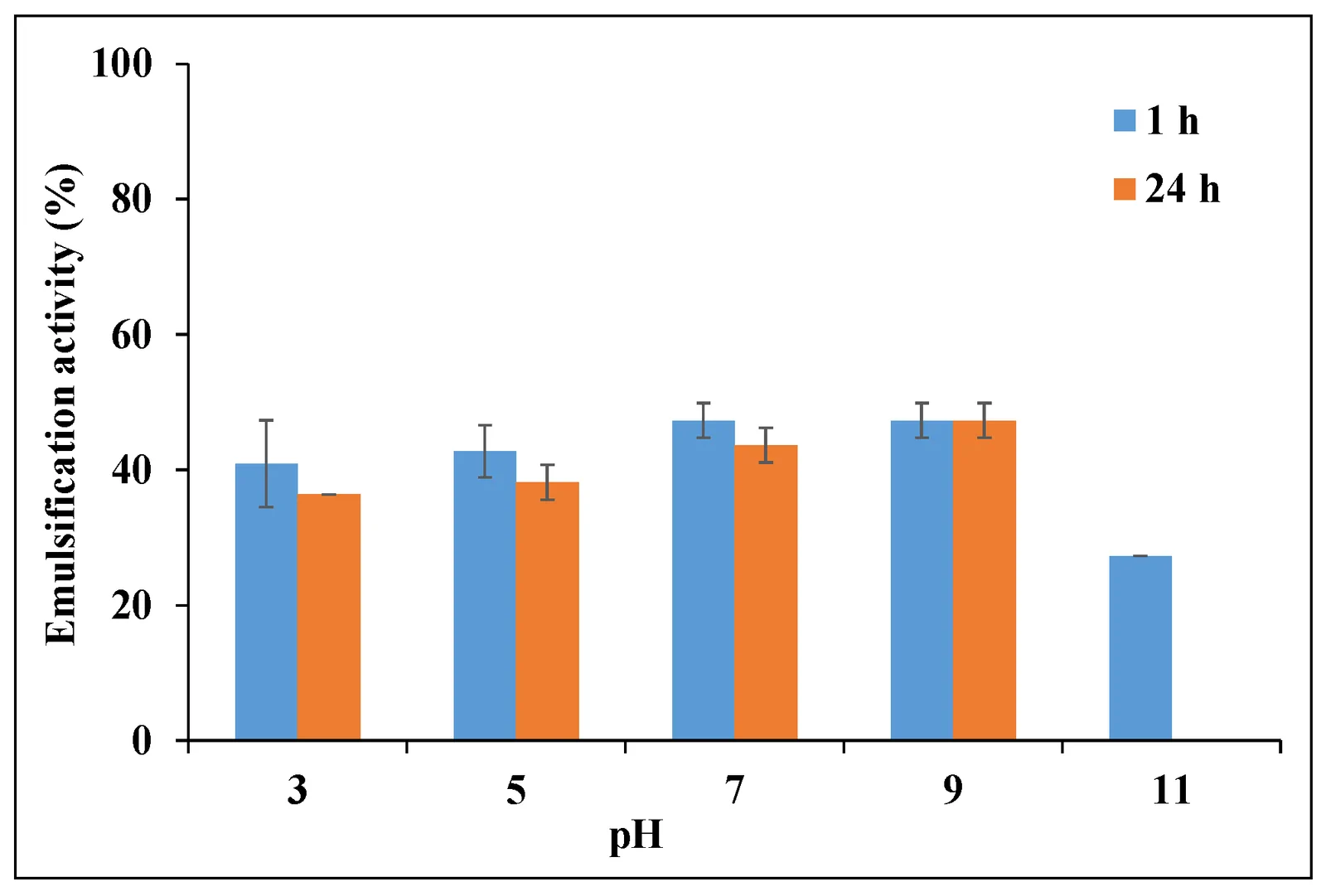
Effect of pH on emulsification activity and emulsion stability of the crude exopolymer (5 g/L) after 1 and 24 h. The results are shown as means ± standard deviation (*n* = 3).

**Figure 3:**
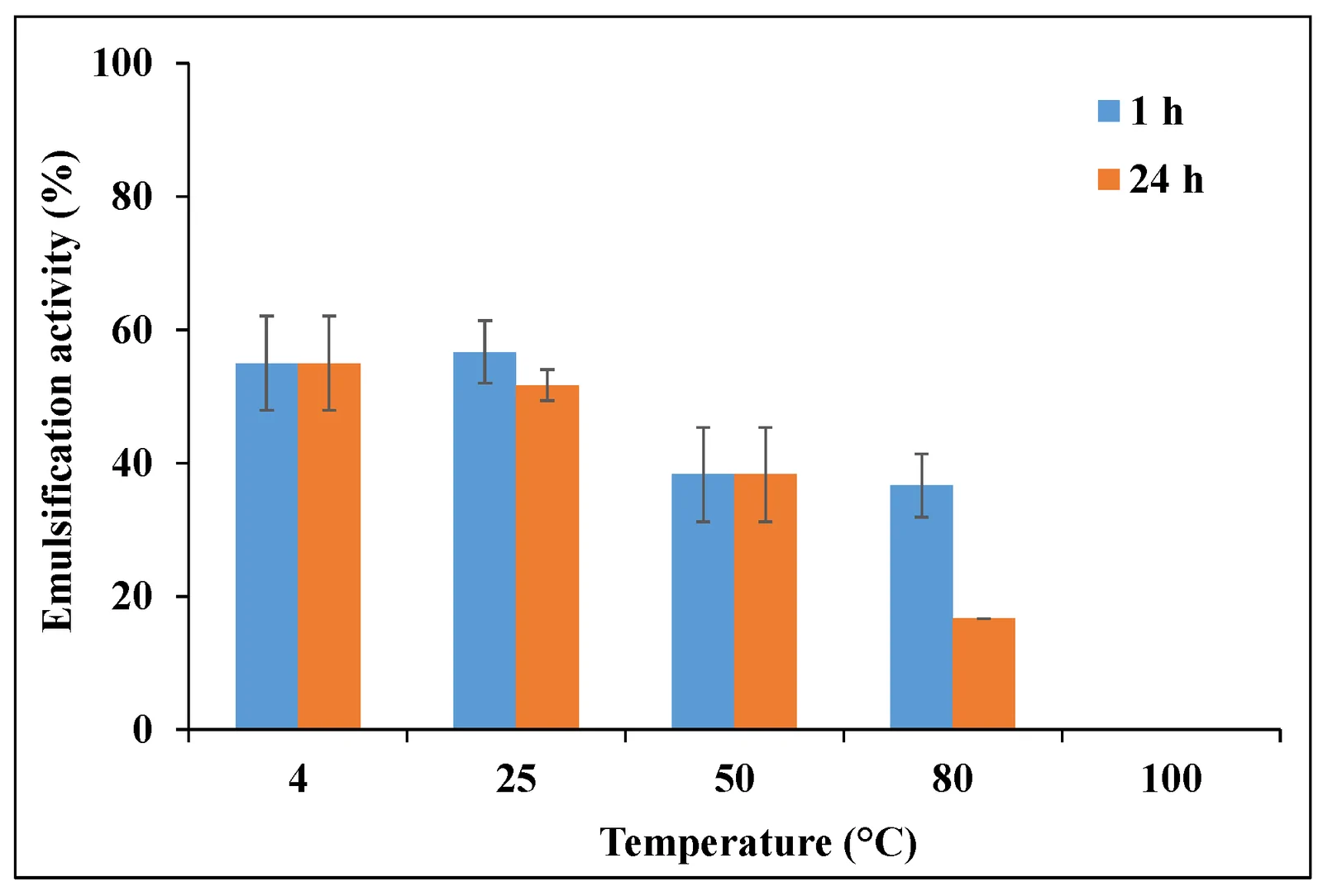
Effect of temperature on emulsification activity and emulsion stability of the crude exopolymer after 1 and 24 h. The results are shown as means ± standard deviation (*n* = 3).

**Figure 4:**
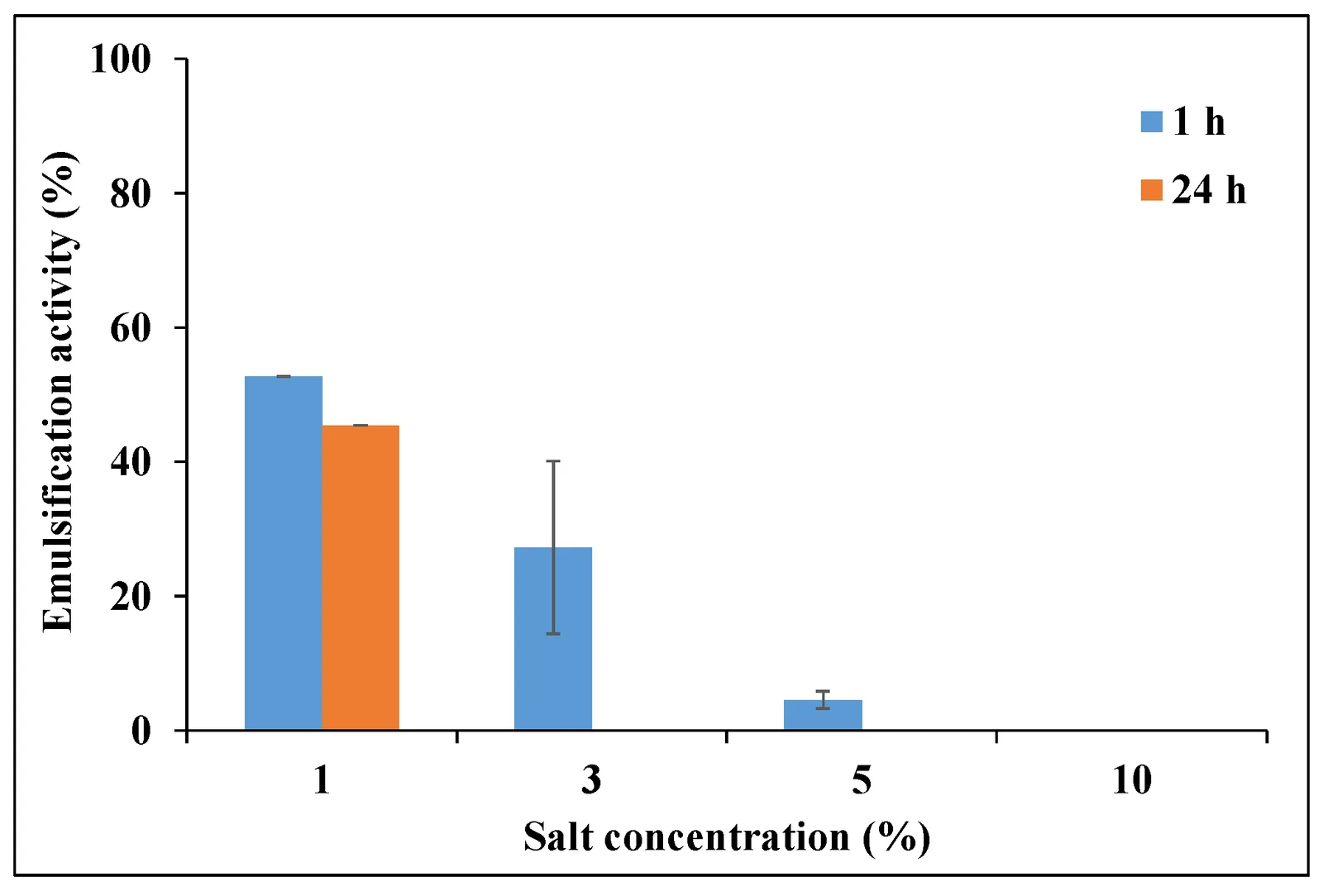
Effect of NaCl on emulsification activity and stability of the crude exopolymer after 1 and 24 h. The results are shown as means ± standard deviation (*n* = 3).

**Figure 5:**
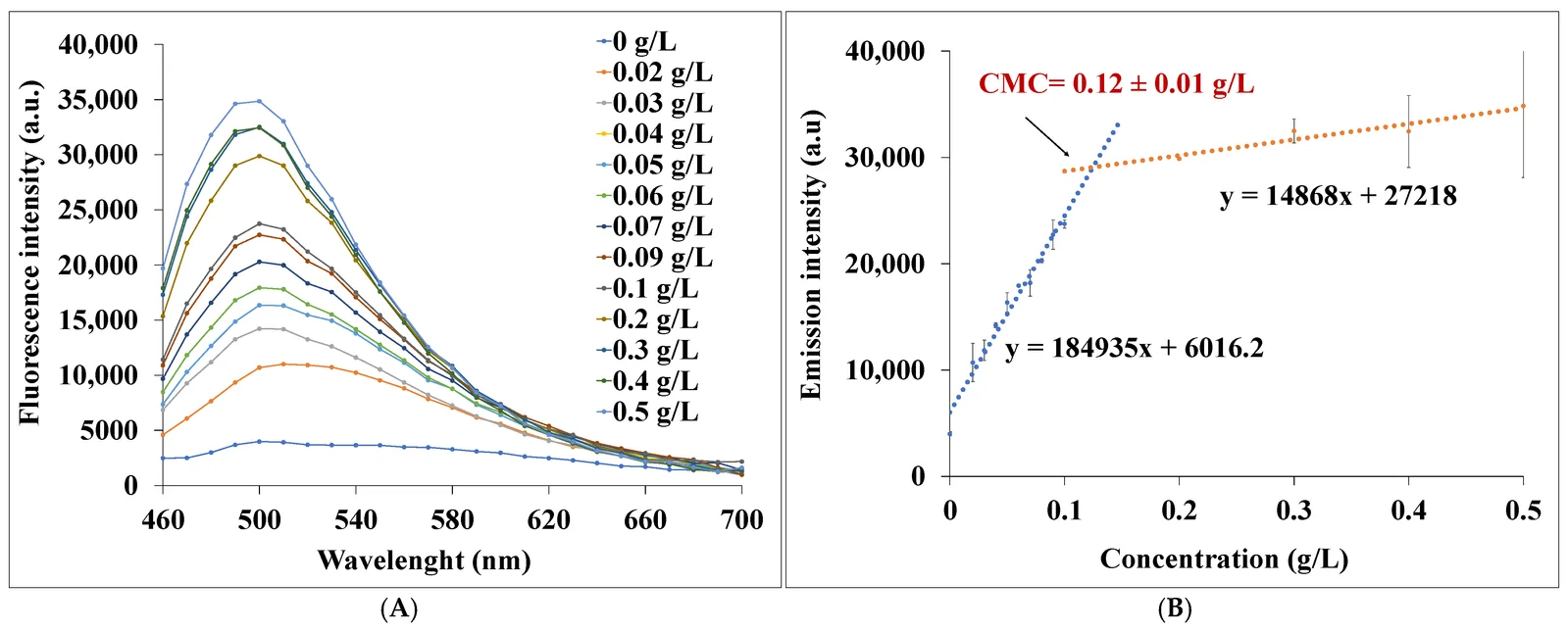
(**A**) Fluorescence intensity spectra of probe curcumin (2 µM, λex = 425 nm). The spectra are averaged over *n* = 2 measurements. (**B**) Dependence of fluorescence intensity of curcumin with different concentration of exopolymer at 500 nm. The dotted blue and orange lines are linear regression lines fitted to two concentration ranges. Their intersection is used to estimate the critical micelle concentration (CMC), 0.12 ± 0.01 g/L.

**Table 1:** Cell growth expressed as cell dry weight (CDW) at given time points and exopolymer production by *R. marinus* DSM 16675 cultivated in media 162 and MB under batch cultivation, or batch cultivation followed by single-cycle fed-batch mode.

Process	Phase	Cultivation Time (h)	M162		MB	
			CDW (g/L)	Exopolymer (mg/L)	CDW (g/L)	Exopolymer (mg/L)
Batch	Batch	0	0.2	0	0.1	0
	Batch	24	0.8	0	0.7	0
Batch + Fed-batch	Batch	0	0.2	0	0.1	0
	End of Batch	12	1.4	0	0.5	0
	24 h fed-batch	36	1.1	3.83	1.0	19.89

**Table 2:** The relative monosaccharide composition (molar ratio) of the purified exopolymer. The amount of glucose is set as 1.

Molar Ratio of the Monosaccharides in the Purified Exopolymer				
Glucose	Galactose	Mannose	Arabinose	Rhamnose
1	1.52	8.44	21.34	26.93

**Table 3:** Amino acid composition of the purified exopolymer.

Amino Acid	Content (g/100 g Exopolymer)
L-Alanine	1.41 ± 0.01
L-Arginine	1.94 ± 0.14
L-Aspartic acid + L-Asparagine	1.47 ± 0.55
L-Cysteine	0.07 ± 0.03
L-Glutamic acid + L-Glutamine	0.91 ± 0.56
Glycine	2.13 ± 0.15
L-Histidine	0.33 ± 0.03
L-Isoleucine	0.74 ± 0.06
L-Leucine	1.59 ± 0.20
L-Lysine	1.71 ± 0.49
L-Methionine	0.51 ± 0.10
L-Phenylalanine	0.77 ± 0.02
L-Proline	0.88 ± 0.05
L-Serine	2.02 ± 0.24
L-Threonine	0.99 ± 0.31
L-Tyrosine	0.62 ± 0.01
L-Valine	1.07 ± 0.01
**Total**	**19.22 ± 0.29**

**Table 4:** Emulsification activity (EA) of extracted crude exopolymer against mineral oil after 1, 24, and 120 h. The results were presented from a single independent experiment (*n* = 1).

Exopolymer (g/L)	EA_1h_ (%)		EA_24h_ (%)		EA_120h_ (%)	
	MB	Control	MB	Control	MB	Control
1	0	40	0	0	0	0
2	36		0		0	
3	36		33		6	
4	50		50		34	
5	57		57		50	

**Table 5:** Dynamic light scattering (DLS) characterization of the crude exopolymer.

Parameter	Value
Z-average (nm)	288.8 ± 63.9
PDI	0.381 ± 0.025
Distribution pattern	Bimodal
Peak size range (nm)	80–120 and 290–374

## Data Availability

The data generated and analyzed during the current study are available from the corresponding author upon request.

## Supplementary Materials

The following supporting information can be downloaded at: https://www.mdpi.com/article/10.3390/ijms27198651/s1.
