# Supplementary material for: Characterization of an Amphiphilic Exopolymer from Rhodothermus marinus DSM 16675 Reveals Its Potential as a Natural Bioemulsifier
Source: Int J Mol Sci. 2026 Sep 27;27(19):8651. doi: 10.3390/ijms27198651 (PMC13664753; doi:10.3390/ijms27198651)
Supplement: Supplementary file 1 [file ijms-27-08651-s001.zip › ijms-4580993-supplementary.pdf]

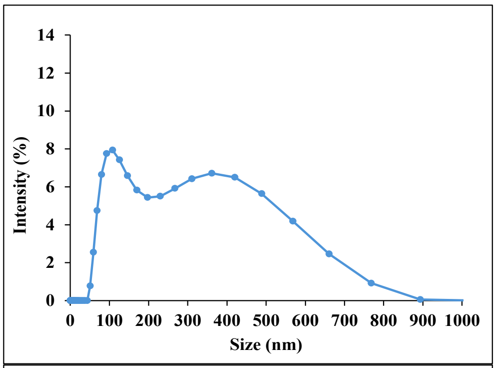

Name	Mean	Standard Deviation	RSD	Minimum	Maximum
Z-Average (nm)	246	-	-	246	246
Polydispersity Index (PI)	0,4091	-	-	0,4091	0,4091
Peak One Mean by Intensity (nm)	118,2	-	-	118,2	118,2
Peak One Area by Intensity (%)	52,8	-	-	52,8	52,8
Peak Two Mean by Intensity (nm)	373,7	-	-	373,7	373,7
Peak Two Area by Intensity (%)	47,2	-	-	47,2	47,2

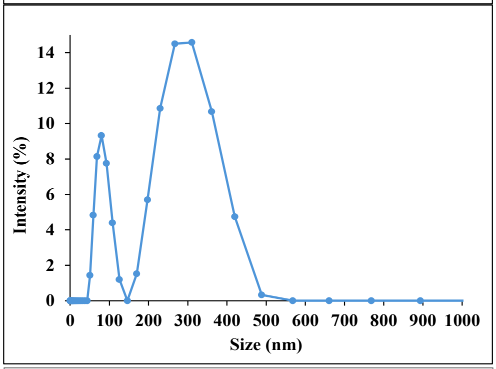

Name	Mean	Standard Deviation	RSD	Minimum	Maximum
Z-Average (nm)	360,5	-	-	360,5	360,5
Polydispersity Index (PI)	0,3619	-	-	0,3619	0,3619
Peak One Mean by Intensity (nm)	290,8	-	-	290,8	290,8
Peak One Area by Intensity (%)	62,92	-	-	62,92	62,92
Peak Two Mean by Intensity (nm)	81,11	-	-	81,11	81,11
Peak Two Area by Intensity (%)	37,08	-	-	37,08	37,08

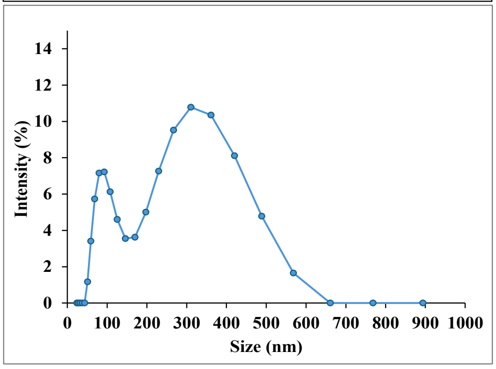

Name	Mean	Standard Deviation	RSD	Minimum	Maximum
Z-Average (nm)	260	-	-	260	260
Polydispersity Index (PI)	0,3729	-	-	0,3729	0,3729
Peak One Mean by Intensity (nm)	311,1	-	-	311,1	311,1
Peak One Area by Intensity (%)	62,39	-	-	62,39	62,39
Peak Two Mean by Intensity (nm)	93,82	-	-	93,82	93,82
Peak Two Area by Intensity (%)	37,61	-	-	37,61	37,61

Figure S1. Dynamic light scattering (DLS) analysis of the exopolymer showing representative intensity-weighted particle size distributions from three independent measurements, with the corresponding hydrodynamic parameters.
